# Supplementary material for: Adult K18-hACE2 mice are suitable for studying intranasal SARS-CoV-2 infection but not direct-contact transmission
Source: Microbiol Spectr. 2025 Oct 20;13(12):e03413-24. doi: 10.1128/spectrum.03413-24 (PMC12671175; doi:10.1128/spectrum.03413-24)
Supplement: Supplemental figures — Figures S1 to S7. [file spectrum.03413-24-s0001.pdf]

Supplementary figure. 1

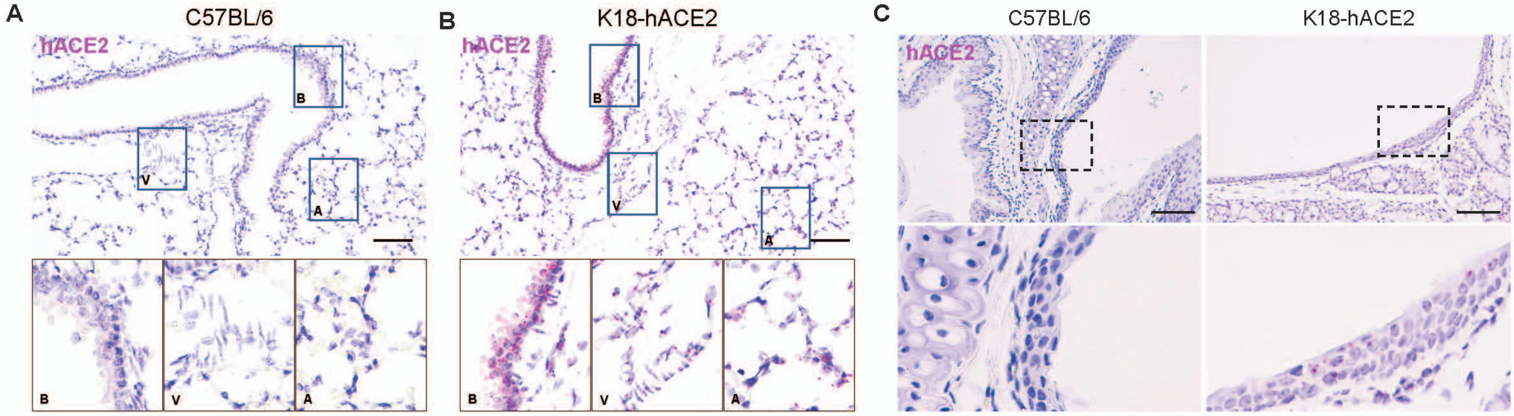

Supplementary figure. 2

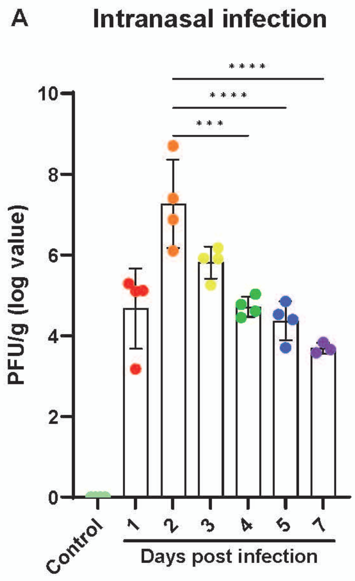

Supplementary figure. 3

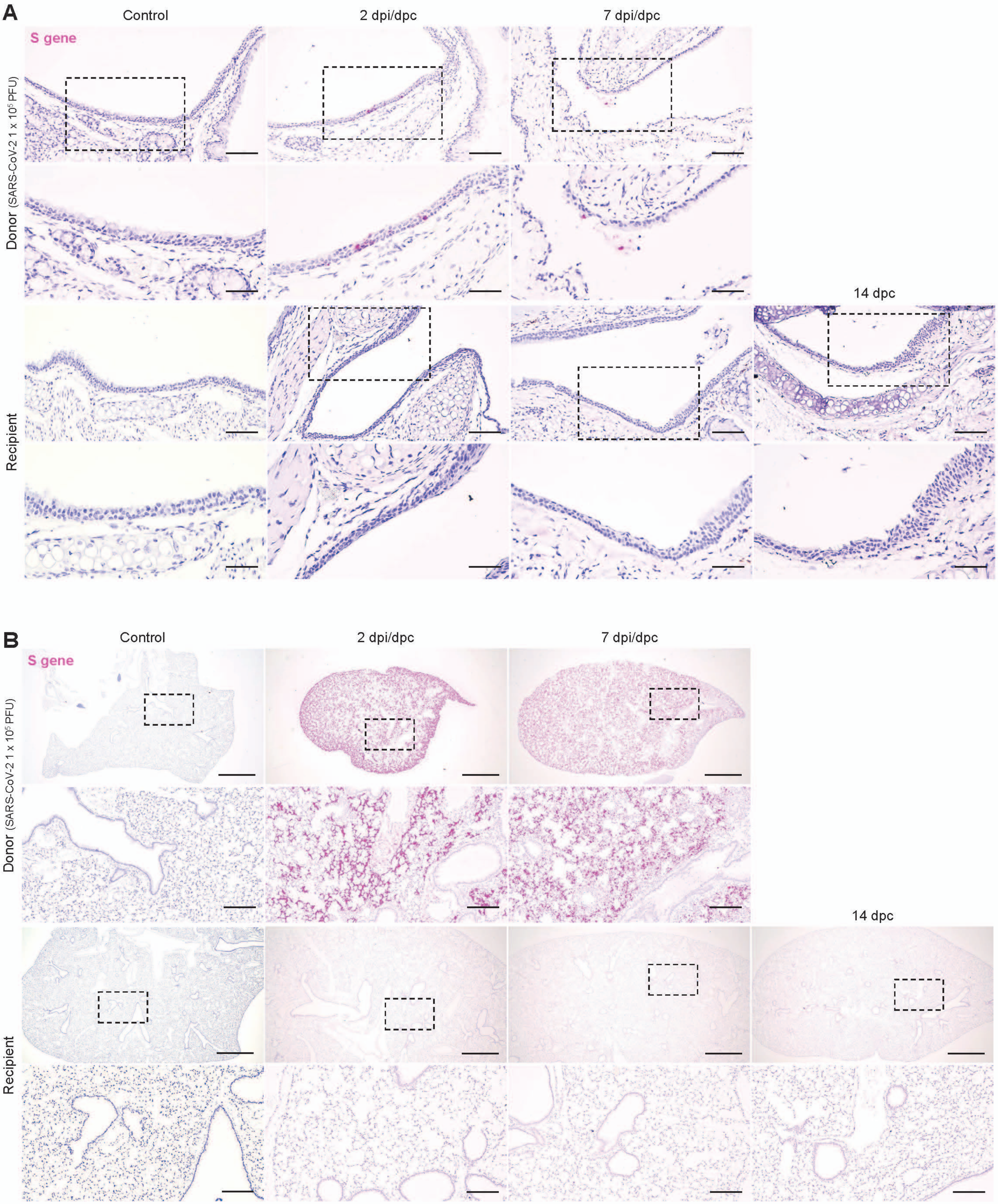

Supplementary figure. 4

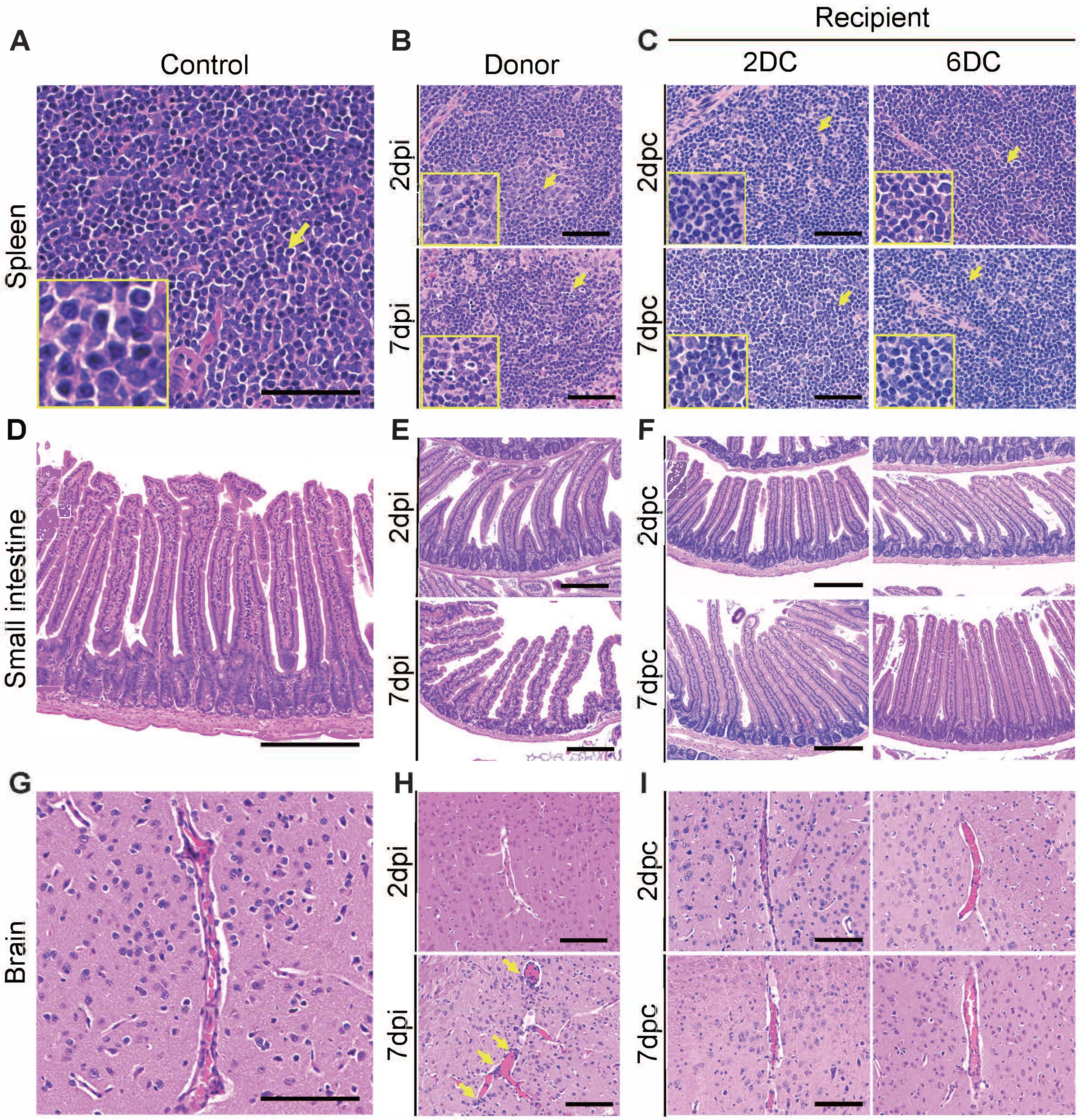

Supplementary figure. 5

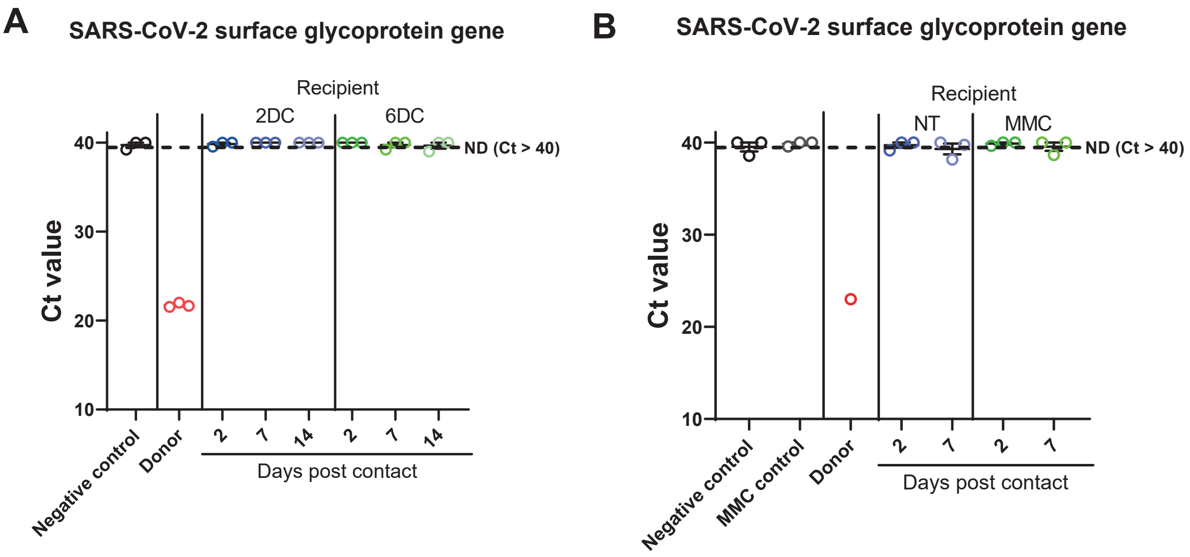

Supplementary figure. 6

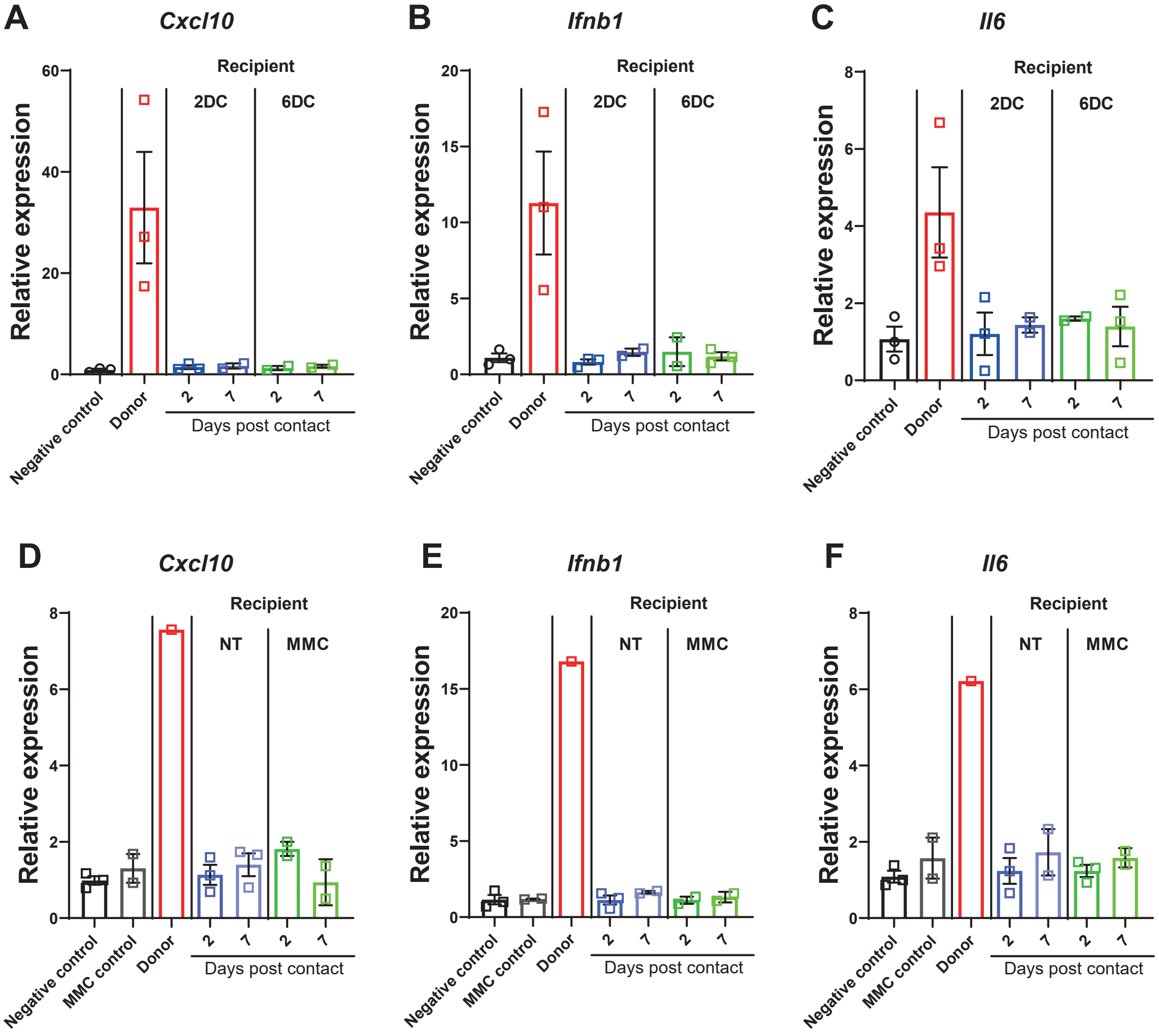

Supplementary figure. 7

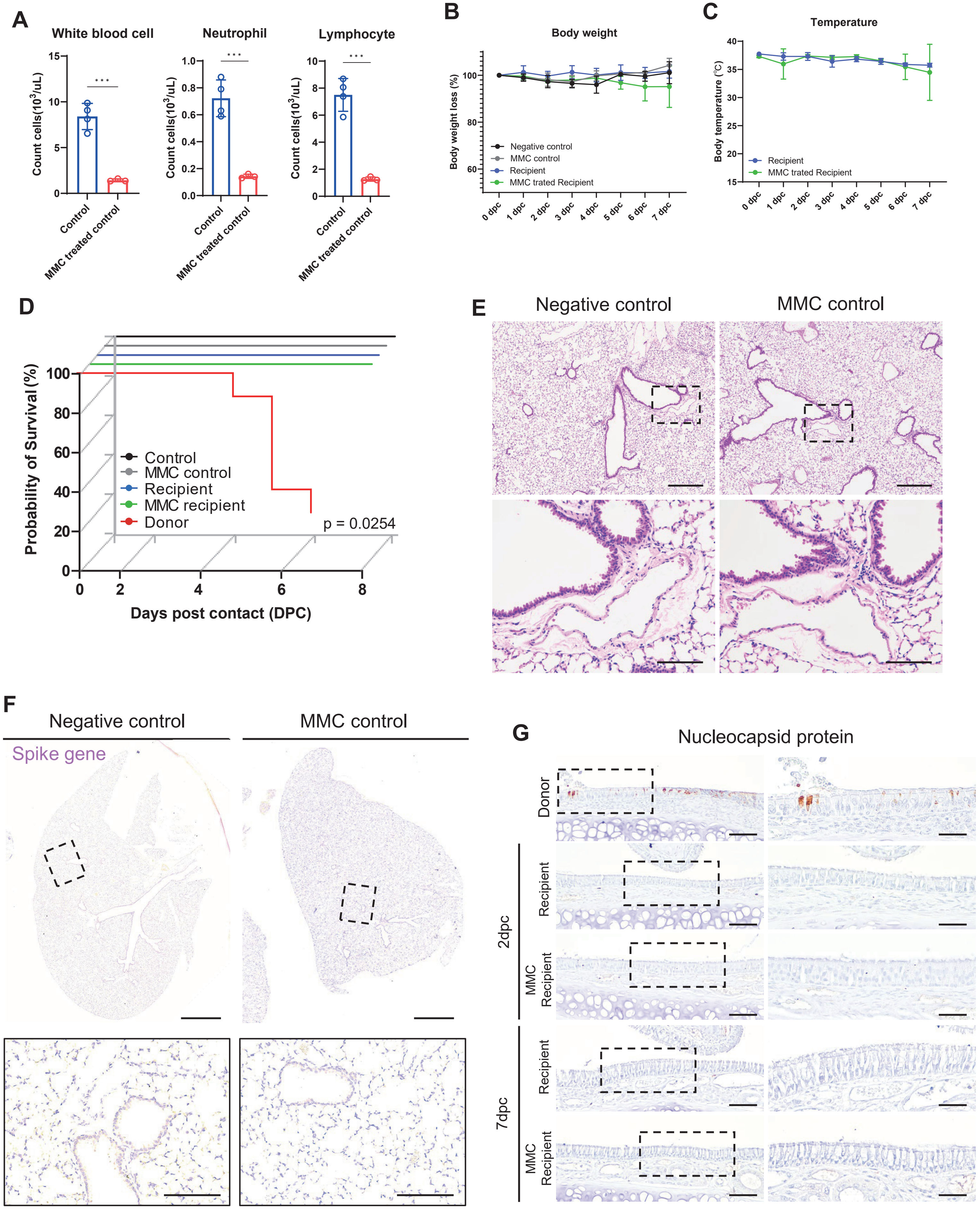

SUPPLEMENTAL FIGURE LEGENDS

Supplemental FIG 1 Expression of human ACE2 RNA in K18-hACE2 transgenic mice. Representative *in situ*-hybridization images of the human (h) *ACE2* gene in the lungs of C57BL/6 (**A**) and K18-hACE2 transgenic (**B**) mice. (C) *ACE2* gene expression in the tracheae of C57BL/6 and K18-hACE2 transgenic mice. Scale bars: 100 μm

Supplemental FIG 2 (**A**) Plaque assay of lung tissues from K18-hACE2 mice (C57BL/6 background) infected with 1x10^{5} PFU of SARS-CoV-2 via intranasal administration. Lung tissues were collected at the indicated time points post-infection, and viral titers were determined by plaque assay. Data are presented as PFU per lung. statistical analysis was performed using one-way ANOVA, and significance was defined as ***P < 0.005 and ****P < 0.001.

Supplemental FIG 3 *S* gene RNA expression in donor and recipient mice lungs and trachea. (**A**) Representative *in situ*-hybridization images of the *S* gene in the tracheae of donor and recipient mice. Scale bars: 100 μm (top); 50 μm (bottom) (**B**) Representative *in situ*-hybridization images for the *S* gene in the lungs of donor and recipient mice. Scale bars: 1 mm (top); 200 μm (bottom)

Supplemental FIG 4 Histopathological changes in extrapulmonary organs from mice infected with SARS-CoV-2 when compared with those in mice subjected to contact transmission. H&E staining of spleen sections for control (**A**), donor (K18-hACE2 mice intranasally infected lungs with SARS-CoV-2) (**B**), and recipients (co-housed with donor mice)

(**C**). The yellow arrows indicate white pulp necrosis. Scale bars: 50 µm. (**D–F**) Small intestine sections for the control (**D**), donor (**E**), and recipient (**F**) groups. Scale bars: 200 µm. (**G–I**) Brain sections for the control (**G**), donor (**H**), and recipient (**I**) groups. The yellow arrows indicate perivascular cuffing. Scale bars: 50 µm

Supplemental FIG 5 Quantification of SARS-CoV-2 surface glycoprotein gene expression in lung tissues from C57BL/6 background mice following the co-housing experiment (**A**) and FVB/NJ background mice subjected to the co-housing experiment with MMC treatment (**B**). RT-qPCR was performed on total RNA extracted from lung tissues of each group (ND: not detected; Ct > 40). Bars represent mean ± SEM from biological replicates.

Supplemental FIG 6 Quantification of mRNA expression levels of *Cxcl10*, *Ifnb1*, and *Il6* in lung tissues from the co-housing experiment in C57BL/6 background mice (**A-C**) and FVB/NJ background mice subjected to the co-housing experiment with MMC treatment (**D-F**). Bars represent mean ± SEM from biological replicates.

Supplemental FIG 7 Signs of contact transmission, clinical symptoms, and respiratory-organ involvement did not occur in FVB-background K18-hACE2 mice, regardless of their immunodeficiency status. (**A**) White blood cell, neutrophil, and lymphocyte counts in intranasally infected FVB-background K18-hACE2 mice and MMC-treated mice, as determined using peripheral blood samples obtained at 7 dpc. P-values were determined by performing two-tailed unpaired Student's *t*-tests (*P < 0.05; **P < 0.01; ****P < 0.001; n.s., not significant). All data are presented as the mean ± s.e.m. (**B**) Body-weight losses in FVB-

background K18-hACE2, MMC-treated, recipient, and MMC-treated recipient mice subjected to donor contact at 2 dpi. (**C**) Body temperatures in MMC-treated, recipient, and MMC-treated recipient mice co-housed with donor contact mice (n = 4–6/dpi). (**D**) Survival rates of FVB-background K18-hACE2 mice intranasally infected with 1 × 10^5 PFUs of SARS-CoV-2 (Donor), recipient mice, MMC-treated recipient mice co-housed with donors, non-infected MMC-treated control mice, and negative-control mice. Survival rate data were analyzed using the Kaplan–Meier method and compared using the log-rank test. (**E**) H&E staining in the lungs of negative-control and MMC-treated control FVB-background K18-hACE3 mice. Scale bars: 500 μm (top rows); 100 μm (bottom rows) (**F**) Representative *in situ*-hybridization images of the *S* gene in lung sections derived from negative-control and MMC-treated control FVB-background K18-hACE3 mice. Scale bars: 1 mm (top); 200 μm (bottom) (**G**) IHC images showing N protein expression in nasal conchae from donor, recipient, and MMC-treated recipient mice. Scale bars: 200 μm (left); 50 μm (right)
